# Advances in Non-Alcoholic Fatty Liver Disease: Pathophysiology, Diagnosis, and Emerging Therapies

**DOI:** 10.3390/life16060915

**Published:** 2026-05-29

**Authors:** Ioannis Katsaros, Dimitrios Schizas

**Affiliations:** First Department of Surgery, Laikon General Hospital, National and Kapodistrian University of Athens, 11527 Athens, Greece; schizasad@gmail.com

## 1. The Shifting Landscape of Fatty Liver Disease

Metabolic dysfunction-associated steatotic liver disease (MASLD) has emerged as one of the most pressing public health challenges, affecting more than 30% of the global adult population and projected to exceed 55% prevalence by 2040 [1]. The recent international consensus renaming non-alcoholic fatty liver disease (NAFLD) to MASLD reflects a fundamental paradigm shift that anchors the disease firmly within its metabolic context, defined by the presence of hepatic steatosis alongside at least one cardiometabolic risk factor [2]. This nomenclature change is not merely semantic, as it reframes how clinicians identify patients at-risk, and the emerging therapies are positioned within a metabolic syndrome framework.

Despite this progress, critical gaps persist. The pathophysiological contributions of the gut microbiome, genetic background, and chronic co-morbidities, such as type 2 diabetes mellitus (T2DM), remain incompletely characterized. Lifestyle and dietary interventions, while accepted as the cornerstone of management, are still poorly optimized with respect to timing, composition, and mechanism. Pharmacological options, though advancing rapidly, remain limited for most patients [3]. This Special Issue, “Advances in Non-Alcoholic Fatty Liver Disease: Pathophysiology, Diagnosis, and Emerging Therapies,” was conceived to address these gaps by bringing together prospective clinical research, experimental animal studies, a randomized controlled trial, and an exploratory microbiome analysis spanning the breadth of contemporary MASLD science.

## 2. Insights from This Special Issue

The first and most clinically expansive contribution to this issue was the prospective study by Rotaru et al., which examined the prevalence, clinical characteristics, and diagnostic predictors of MASLD in a cohort of 387 lean individuals, with particular focus on those with concurrent IBD [4]. The study reported a MASLD prevalence of 34.1% among lean subjects overall, rising to 46.3% in those with IBD, a significant difference that underscores IBD as an independent risk factor for hepatic steatosis even in the absence of obesity. Patients suffering from both IBD and MASLD exhibited greater hepatic steatosis, which is consistent with the pivotal role of gut–liver axis disruption in MASLD pathogenesis. Intestinal barrier dysfunction in IBD facilitates the translocation of bacterial products and endotoxins into the portal circulation, triggering hepatic inflammation through toll-like receptor activation and hepatic stellate cell stimulation, thus promoting fibrogenesis and disturbing lipid metabolism.

A focused commentary by Scarlata [5] offered an important critical perspective on the Rotaru et al.’s findings, drawing on data from their own IBD cohort in which the IBD-MASLD population was significantly more overweight and showed higher frequencies of cardiometabolic parameters, including type 2 diabetes and hypertension, compared with patients classified under the previous NAFLD nomenclature. This observation serves as a timely reminder that while the MASLD definition formally accommodates a lean phenotype, in high-risk populations such as those with IBD, the new nomenclature may predominantly capture overweight and obese individuals. The authors called for longitudinal studies in large IBD cohorts enriched with genomic and transcriptomic analyses capable of providing prognostic signatures for patient stratification. The bidirectional communication between the gut and liver mediated through the portal circulation, encompassing gut-derived microbial products, altered bile acid signaling, and inflammatory mediators, warrants systematic investigation within this growing patient population.

The only randomized controlled trial in this Special Issue, conducted by Ozlu Karahan et al. [6], investigated the effects of a 16:8 time-restricted eating (TRF) protocol on hepatic steatosis, fibrosis, serum fibroblast growth factor (FGF)-21, and autophagy markers in 48 overweight or obese patients with MASLD. The addition of the TRF protocol produced significantly greater reductions in CAP, body weight, BMI, waist circumference, body fat mass, body fat percentage, and total cholesterol compared with energy restriction alone. A key mechanistic finding was the significant increase in autophagy-related protein 5 (ATG-5) levels observed exclusively in the TRF group, while Beclin-1 levels remained unchanged in both groups. This trial nonetheless establishes meaningful data supporting the clinical prescription of TRF as an adjunct dietary strategy in overweight and obese MASLD patients.

The experimental study by Sotiropoulou et al. [7] represents the most mechanistically detailed contribution to this issue, examining the hepatoprotective effects of quercetin, silibinin, and crocetin in a high-fat diet-induced mouse model of MASLD. Ninety-five C57BL/6J mice were randomized to receive low or high doses of each following twelve weeks of high-fat feeding to establish hepatic steatosis. All three natural polyphenols significantly reduced steatohepatitis prevalence compared to controls, with the most significant effects in the crocetin and quercetin groups. The study’s distinctive contribution is based on its immunohistochemical characterization of CD36 and perilipin-3 (PLIN3) expression as key mediators of the therapeutic response. CD36 was significantly downregulated by silibinin at low doses, an effect consistent with its known anti-lipogenic properties and its capacity to attenuate fatty acid influx. PLIN3, a lipid droplet-associated protein involved in the formation and stabilization of intracellular lipid droplets, was significantly upregulated in the quercetin, low-dose silibinin, and high-dose crocetin groups. This pattern suggests that upregulation of PLIN3 promotes a protective lipophagy pathway, facilitating the safe sequestration and autophagic clearance of intrahepatic lipids and thereby ameliorating hepatocellular stress.

The most geographically and methodologically ambitious contribution was the multicenter study by Suarez et al. [8], which recruited 214 patients with T2DM from five distinct geographical regions of Argentina to characterize the interactions between gut microbiome signatures, the PNPLA3 rs738409 genetic polymorphism, and clinical risk factors for MASLD. With a MASLD prevalence of 77.9% the study identified marked regional variations in gut microbiota diversity and core microbiome composition. Analysis established the PNPLA3 GG genotype as an independent risk factor for elevated FIB-4 scores, while paradoxically acting as a protective factor against higher HbA1c, fasting plasma glucose, and total cholesterol levels. From a microbiome perspective, the Negativibacillus genus was exclusively detected in the core microbiota of both MASLD patients and PNPLA3 GG carriers, with the strength of this association modified by FIB-4 score remaining significant in intermediate- and high-risk fibrosis groups. The Catenibacterium genus, associated with pro-inflammatory and pro-fibrogenic properties, was uniquely observed in patients with FIB-4 > 2.67. Conversely, short-chain fatty acid-producing bacteria, including members of the Eubacterium genus, were linked to the absence of MASLD and lower fibrosis risk, consistent with their established roles in improving insulin sensitivity and intestinal gluconeogenesis. These findings validate the principle that microbiota biomarkers for MASLD risk stratification should be the basis for relevant diagnostic and therapeutic strategies.

## 3. Synthesis and Future Directions

Overall, the five contributions to this Special Issue highlight the heterogeneity of MASLD and the necessity of shifting to an individualized approach for its diagnosis and management. Several themes emerge from these studies.

First, the limitations of BMI as the primary diagnostic and risk-stratification tool for fatty liver disease are reaffirmed across multiple studies. Lean MASLD, driven by visceral adiposity, dyslipidemia, insulin resistance, and chronic inflammation rather than overt obesity, demands alternative metrics such as CUN-BAE, waist circumference, and metabolic criteria-based definitions [2,4]. The MASLD framework, by linking diagnosis to cardiometabolic criteria, provides a more inclusive and biologically meaningful classification that better captures at-risk individuals across the BMI spectrum.

Moreover, the gut–liver axis emerges as a unifying pathophysiological concept across the issue. Whether examined through the lens of IBD-associated intestinal permeability, [4,5], the autophagic responses modulated by fasting cycles [6], or the microbial signatures associated with fibrosis and genetic susceptibility [8], dysbiosis and its downstream hepatic consequences appear critical to MASLD progression. Future studies integrating gut microbiota profiling, metabolomics, and genomic data in large prospective cohorts will be essential to translate these observations into validated biomarkers.

Both dietary and pharmacological strategies targeting autophagy, lipid metabolism, and oxidative stress hold genuine therapeutic promise. The TRF protocol’s induction of ATG-5 and reduction in FGF-21 in a clinical trial setting [6], alongside the PLIN3 upregulation and CD36 modulation achieved by natural polyphenols [7], converge on autophagy and lipophagy as targets deserving further investigation. These findings are particularly relevant given the emerging role of autophagy impairment in the transition from simple liver steatosis to metabolic dysfunction-associated steatohepatitis (MASH) and fibrosis.

Finally, the role of concurrent diseases in amplifying MASLD risk and altering its phenotypic expression demands dedicated multidisciplinary attention. Hepatologists, endocrinologists, and dietitians must collaborate to properly diagnose and treat MASLD, specifically in challenging patients.

## 4. Conclusions

The Special Issue “Advances in Non-Alcoholic Fatty Liver Disease: Pathophysiology, Diagnosis, and Emerging Therapies” has brought together a diverse and complementary collection of research that advances our understanding of MASLD across its spectrum. We are grateful to all contributing authors for their rigorous and innovative work, to the reviewers who strengthened these manuscripts through their expert critique, and to the editorial team of *Life* Journal for their support throughout this process. The field of MASLD continues to evolve at remarkable speed, and it is our hope that the insights gathered in this issue will contribute meaningfully to the next generation of clinical and translational research directed at this global health challenge.
